# Artificial intelligence-driven personalized dietary recommendations for gastric cancer high-risk populations: a narrative review

**DOI:** 10.3389/fnut.2026.1802970

**Published:** 2026-03-26

**Authors:** Jiahao Chen, Tianyuan Sun, Jiayi Zhang, Jiarong Huang, Tianci Chen, Yihui Weng, Hanting Xiang, Zhebin Dong, Zhonting Huang, Xianlei Cai, Chao Liang, Miaozun Zhang, Weiming Yu

**Affiliations:** Department of Gastrointestinal Surgery, The Affiliated Lihuili Hospital of Ningbo University, Ningbo, China

**Keywords:** artificial intelligence, gastric cancer, nutritional intervention, personalized diet, risk stratification

## Abstract

This review summarizes the current applications of artificial intelligence (AI) in providing personalized dietary recommendations, and explores its potential applicability to populations at high risk for gastric cancer. Currently, there are no direct intervention trials for gastric cancer patients. However, evidence from metabolic diseases (like diabetes and obesity) shows that AI-driven dietary interventions could be beneficial. This approach may offer translatable benefits for cancer prevention. First, the paper elaborates on the severe incidence of gastric cancer and the limitations of traditional preventive measures, emphasizing the necessity of developing precise and efficient intervention strategies. Subsequently, it systematically outlines methods for identifying high-risk populations and risk stratification (including pathological basis, biomarkers, and genetic risks), as well as the close relationship between dietary patterns (protective and risky) and gastric cancer risk, with a particular focus on the interaction between diet and the gastric microbiome (especially *Helicobacter pylori*). The core section analyzes the technical principles of AI-driven personalized nutritional interventions (such as machine learning and deep learning) and their practical effects in improving chronic diseases like blood glucose control and obesity management, while looking forward to the potential of integrating AI with multi-omics data. In addition, the paper extends the discussion to the extended applications of AI in improving screening adherence, assisting endoscopic diagnosis, and clinical decision support systems. Finally, the paper points out current challenges such as technical interpretability, data privacy, population differences, and clinical validation, and proposes prospects for future research directions.

## 1 Background and research significance

Gastric cancer is the fifth most common cancer globally and the third leading cause of cancer-related deaths (1). Risk factors for the disease include *Helicobacter pylori* infection, age, high-salt diet, and insufficient intake of fruits and vegetables. Although the overall incidence has declined in recent years, there remains a high incidence trend among certain high-risk populations. Early screening and preventive intervention are crucial for reducing gastric cancer-related mortality (2); however, there is a general lack of efficient and individualized risk assessment and intervention methods.

In this context, applying AI to deliver tailored dietary guidance for individuals at high risk of gastric cancer has emerged as a key area of research interest. By integrating biomarkers, lifestyle data, and machine learning algorithms, this strategy aims to provide precise and actionable nutritional recommendations, thereby improving metabolic health and reducing cancer risk (3). This approach not only holds promise for enhancing intervention effectiveness but also offers a new model for the long-term management of chronic diseases.

## 2 Identification of high-risk populations and risk stratification

### 2.1 Pathological basis and risk assessment tools

The development of gastric cancer begins with chronic inflammation, progressing through chronic atrophic gastritis, intestinal metaplasia, dysplasia, and ultimately invasive carcinoma— a multi-stage process known as the “Correa cascade” (103). Chronic atrophic gastritis is an early lesion of gastric cancer, commonly caused by *Helicobacter pylori* infection-induced multifocal atrophic gastritis (type B gastritis) or autoimmune-induced autoimmune gastritis (type A gastritis) (4). The Gastritis OLGA system can be used to guide and prevent the progression of atrophic gastritis to gastric cancer (5). Intestinal metaplasia refers to the replacement of normal gastric epithelium in the antrum or acid-secreting mucosa by intestinal epithelium, including intestinal epithelial cell types such as Paneth cells, goblet cells, and absorptive cells (6). Nuclei in low-grade dysplasia are usually round with a high nuclear-cytoplasmic ratio, prominent nucleoli, and preserved polarity (7). Dysplasia can be classified into low-grade dysplasia (LGD) and high-grade dysplasia (HGD). LGD is characterized by rod-shaped nuclei with a low nuclear-cytoplasmic ratio and preserved polarity, while HGD exhibits loss of cellular polarity (7).

In terms of screening and early diagnosis, the G-score model can be applied to large-scale combined screening for upper gastrointestinal cancers. Compared with traditional methods, this model improves the detection rate of malignant lesions while reducing testing costs (8). The Gastric Cancer Risk Assessment Program (GRAPE) can also be used for early gastric cancer detection. GRAPE is significantly superior to radiologists, as it can detect GC cases initially missed by radiologists, enabling earlier diagnosis during disease follow-up (9). Pathological assessment using AI models can not only distinguish high-risk from low-risk patients but also indicate the tumor microenvironment status and potential drug sensitivity, laying the foundation for “precision medicine” (10). For advanced patients, clinical prognostic models integrated with Patient-Reported Outcomes (PROs) can be used to assess survival, and genetic models or biomarkers can be referenced to identify treatment opportunities (11, 12).

### 2.2 Biomarkers

Clinically common high-risk biomarkers include precancerous lesions such as chronic atrophic gastritis, intestinal metaplasia, and dysplasia (13). These lesions can be confirmed by gastroscopy combined with biopsy and serve as important bases for formulating individualized follow-up plans.

Serological tumor markers: CEA, CA19-9, CA72-4, etc., have been widely used in clinical practice (14). circAHSA1 is significantly upregulated in the serum of gastric cancer patients, and its combined use with traditional markers such as CEA can improve diagnostic accuracy (15). For early diagnosis, serum CCR5, CCL5, PDGF-BB, and EphA7 are significantly elevated, among which PDGF-BB has the highest diagnostic value (16). ctDNA and liquid biopsy technology can be used for early gastric cancer screening, postoperative minimal residual disease detection, and formulating personalized treatment plans for gastric cancer patients (17, 18). In addition, low expression of circulating precursor miR-488 and miR-488-5P is associated with poor prognosis in gastric cancer patients, and precursor miR-488 is an independent predictor of overall survival (19).

Histological biomarkers: Microsatellite instability (MSI) and EBV-positive gastric cancer often express immune checkpoint molecules such as PD-L1 and VISTA; chromosome instability tumors commonly exhibit overexpression of receptor tyrosine kinases such as EGFR, FGFR2, HER2, and MET; genomic stability tumors frequently show alterations in Claudin 18.2 (20).

### 2.3 Genetic and familial risks

Some gastric cancers have a clear genetic background, such as Hereditary Diffuse Gastric Cancer (HDGC), Gastric Adenocarcinoma and Proximal Polyposis of the Stomach (GAPPS), and Familial Intestinal-Type Gastric Cancer (FIGC) (21). HDGC is an autosomal dominant cancer syndrome mainly caused by germline mutations in the CDH1 and CTNNA1 genes (22). Studies have shown that carriers of CTNNA1 truncating mutations have an eight-fold higher risk of developing Diffuse Gastric Cancer (DGC) than non-truncating carriers (23). Among families with a family history of HDGC-related death, 76.5% of individuals who underwent prophylactic gastrectomy were diagnosed with occult DGC (24). GAPPS is a recently described rare autosomal dominant genetic disease caused by point mutations in the APC gene promoter1B (25). The genetic basis of FIGC has not been fully elucidated, but it may be associated with mutations in the PALB2 and BRCA2 genes (26). For individuals with a clear family history, genetic counseling and multigene testing are essential (27).

## 3 Relationship between dietary patterns and gastric Cancer risk

### 3.1 Protective dietary pattern

Multiple studies consistently show that protective dietary patterns are usually rich in fruits, vegetables, whole grains, and legumes (28). Among individuals with light alcohol consumption (less than two servings of alcohol per day), adequate folate intake is beneficial for reducing the risk of gastric cancer (29). Major dietary sources of folate include leafy green vegetables such as cabbage, broccoli, and lettuce, as well as animal liver, eggs, and milk (30). Polyunsaturated fatty acids (PUFAs), mainly found in deep-sea fish, are associated with a lower risk of gastric cancer in populations with higher intake (31). Whole-grain diets are also related to the risk of gastric cancer; high intake of refined grains not only reduces the risk of gastric cancer but also lowers the risk of colorectal cancer, pancreatic cancer, and esophageal cancer (32).

Regarding specific dietary patterns: The Mediterranean diet is plant-based with olive oil as the main fat source. A meta-analysis showed that strict adherence to this dietary pattern can reduce the risk of gastric cancer by up to 58% (33). The DASH diet (Dietary Approaches to Stop Hypertension) was shown in an Iranian case–control study to reduce the risk of gastric cancer by 54% in highly compliant individuals (OR = 0.46) (34). High adherence to a healthy/prudent dietary pattern (rich in vegetables, fruits, fish, etc.) can reduce the risk of gastric cancer by 22%. A low-inflammatory index diet, which aims to reduce intake of saturated fats and refined carbohydrates, can lower the risk of gastric cancer by 32% (35).

### 3.2 Risky dietary patterns

In contrast, “risky” dietary patterns are characterized by high intake of fat, sugar, salt, alcohol, and red meat (36). Long-term consumption of fried foods, as high-risk carcinogens, can significantly increase the risk of gastric cancer (OR = 1.52) (37). High-sugar diets can affect insulin secretion levels, thereby influencing the Dietary Insulin Index (DII) and Dietary Insulin Load (DIL) (38). An Afghan case–control study showed that individuals with DIL in the highest tertile had a 3.41-fold higher risk of gastric cancer than those in the lowest tertile (39). Heavy alcohol consumption (>42 grams/day) and pickled fish intake (RR = 1.56 when comparing the highest and lowest categories) are both significantly positively correlated with gastric cancer risk (36). A study in Hanzhong, China found that frequent consumption of fresh meat (OR = 3.0), processed meat (OR = 3.3), dairy products (OR = 3.1), and fish (OR = 3.1) all significantly increased the risk of gastric cancer (40). Data from a Korean study showed that high-salt pickles accounted for 16.0% of gastric cancer cases, making them the primary preventable factor (41).

### 3.3 Interaction between diet and microbiome

Emerging research focuses on how diet affects cancer risk by regulating the gastric microbiome. The gastric mucosa of gastric cancer patients is often enriched with bacterial genera such as Lactobacillus, *Escherichia coli*-Shigella, Nitrospira, Burkholderia, and uncultured Lachnospiraceae. Among these, Nitrospira is present in all gastric cancer patients but not in patients with chronic gastritis (42), suggesting it may be a high-risk factor for gastric cancer. A high-nitrite diet can increase the number of Nitrospira in the stomach, thereby elevating nitric oxide (NO) levels, which play a key role in various cancers (43). *Helicobacter pylori* is a known high-risk factor for gastric cancer, and the relationship between diet and its infection is discussed in detail below.

High-risk dietary factors for *Helicobacter pylori* infection: Animal experiments have shown that a high-salt diet not only damages the gastric mucosal barrier and downregulates the expression of mucin in surface mucous cells (44, 45), but also leads to enhanced proliferation of gastric mucosal cells, lipid peroxidation, and histological changes such as intestinal metaplasia (46), thereby promoting *Helicobacter pylori* colonization. In addition, high-carbohydrate/sweet food patterns (47)and high-red meat patterns are also high-risk factors for *Helicobacter pylori* infection (48).

**Figure 1:**
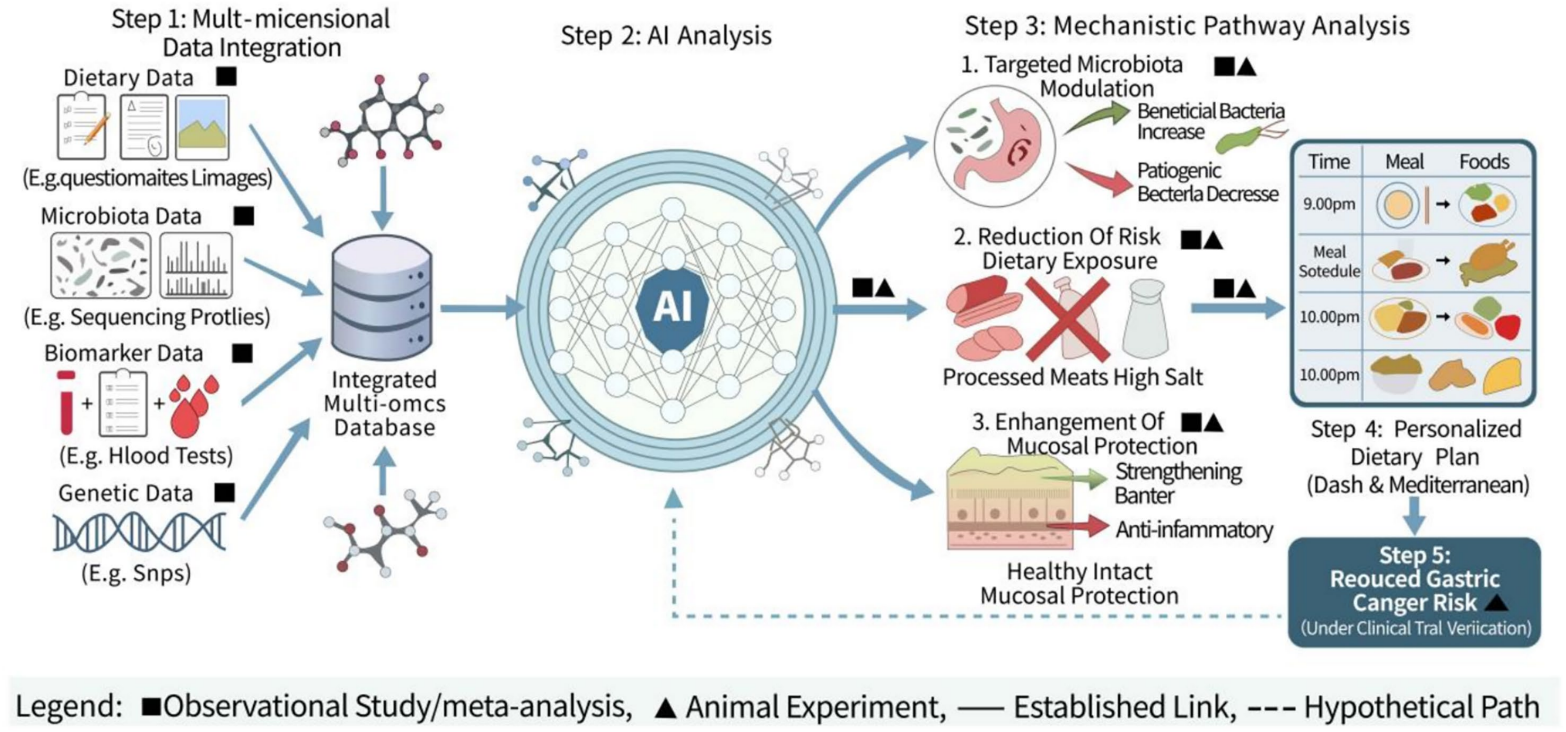
The diet-microbiota-risk axis and personalized AI-dietary intervention framework for gastric cancer.

Protective dietary factors for *Helicobacter pylori* infection: Daily fiber intake higher than the Recommended Nutrient Intake (RNI) can reduce the infection rate (49). Consumption of chili (48), honey, and green/black tea can also decrease the *Helicobacter pylori* infection rate (50). Mouse model studies have shown that bovine milk glycoproteins can reduce the colonization of *Helicobacter pylori* in the stomach (51). Cruciferous vegetables have bactericidal effects against *Helicobacter pylori* (52) (Figure 1).

## 4 Application of artificial intelligence in personalized dietary interventions

### 4.1 Technical principles and implementation pathways

AI-generated personalized dietary recommendations mainly rely on machine learning (ML) algorithms, which integrate multiple inputs such as glycemic dynamics, gut microbiota composition, and self-reported data to output customized dietary plans. Common technologies include traditional machine learning (ML), deep learning (DL), and hybrid systems combined with the Internet of Things (IoT)^3^. ML generates personalized recommendations using data such as genotype, biomarkers, dietary habits, and physiological responses (53). DL uses variational autoencoder networks to process personal information, facilitates recurrent neural networks to generate dietary sequences, and constructs weekly meal plans for users (54). The application of Large Language Models (LLMs, such as ChatGPT) and Natural Language Processing (NLP) technology can provide users with personalized dietary advice, exercise plans, and psychological support (55, 56). Although wearable IoT systems have been used to monitor nutritional data, they lack molecular data reflecting physical reactions and metabolic characteristics, thus limiting their application (57).

### 4.2 Practical intervention effects

Existing systems optimize carbohydrate selection by analyzing individuals’ glycemic responses to food, and have shown effectiveness in improving symptoms in patients with diabetes and irritable bowel syndrome (IBS), such as a 39% reduction in IBS symptom severity and a 72.7% diabetes remission rate^3^. AI-based personalized Postprandial Glycemic Response (PPGR) prediction tools help improve glycemic responses in high-risk populations for diabetes, thereby reducing the adverse consequences of long-term and repeated exposure to hyperglycemia (58). In the field of obesity and overweight, ML algorithms can be used to predict the relationship between obesity-related diets and the microbiome, thereby guiding dietary adjustments (59). In addition, AI can automatically estimate nutrient intake by identifying food images, reducing the errors of traditional self-reported dietary records, significantly improving assessment efficiency, and assisting in nutritional diagnosis (60, 61). These findings demonstrate AI’s efficacy in metabolic disease management. However, direct extrapolation to GC prevention is premature; while biologically plausible, cancer-specific trials are needed to confirm benefit.

### 4.3 Integration of multi-omics data and artificial intelligence

**Figure 2:**
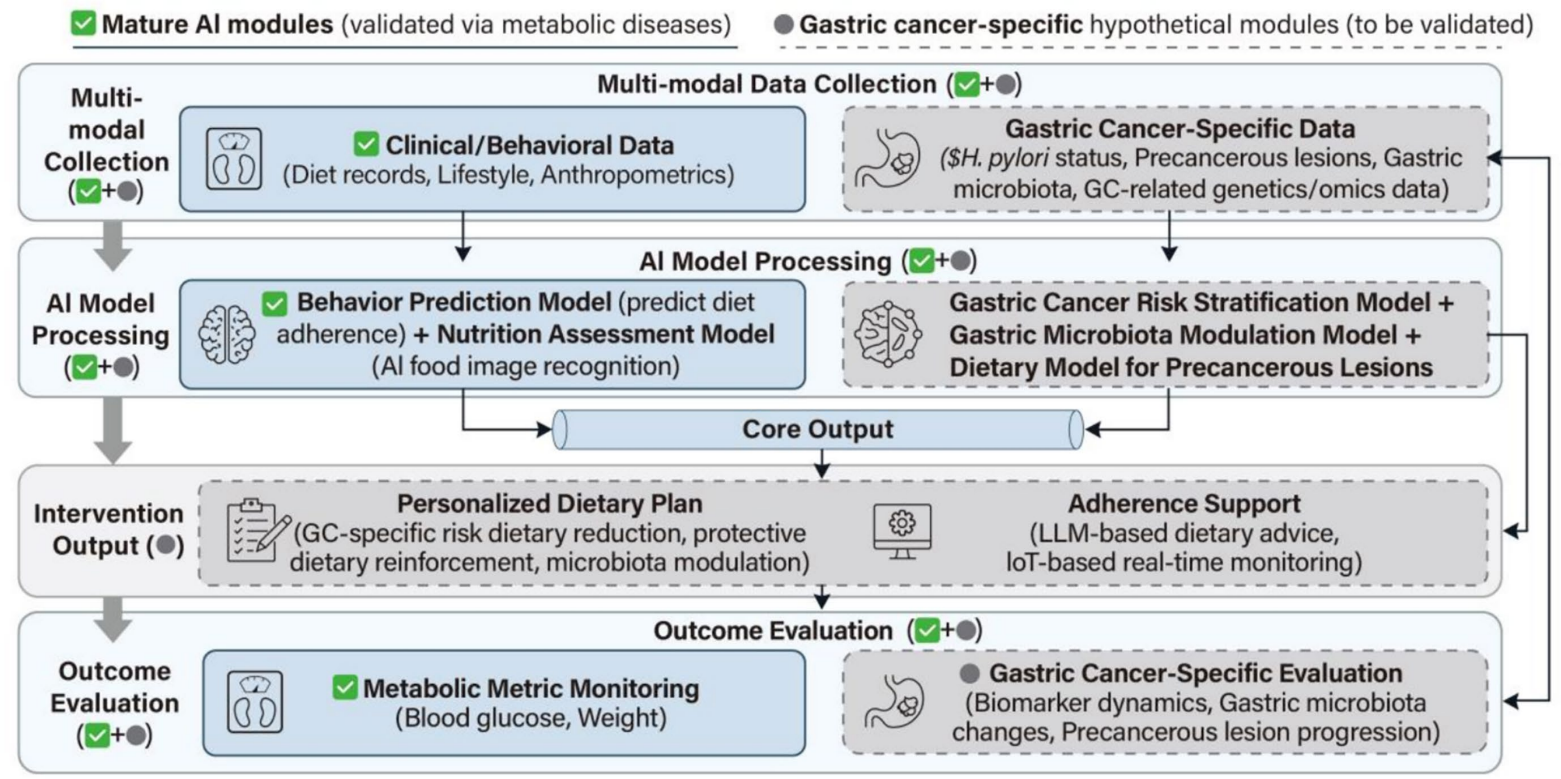
AI-driven personalized dietary intervention workflow for gastric cancer high-risk populations.

The integration of multi-omics technology and AI has a profound impact on nutritional science. Large-scale clinical trials have shown that multi-omics-driven personalized nutritional interventions are superior to conventional methods in weight management, blood glucose control, and dietary adherence (62), and can reveal individualized molecular mechanisms of related chronic diseases. AI can integrate multi-omics data to provide precise strategies for risk early warning and nutritional intervention of specific diseases (63). The AdaptaFood system is an application of AI in personalized diets, which can generate recipes consistent with users’ biological backgrounds and preferences based on knowledge graphs (64) (Figure 2).

## 5 Behavioral intervention and improvement of screening adherence

### 5.1 Network information-driven behavioral change models

A study conducted in China adopted a network health education model based on the “Behavior Change Wheel (BCW)” theory to intervene 260 high-risk individuals for one year. The results showed that after intervention, the gastroscopy rate, cancer cognitive level, and healthy dietary behavior of the experimental group were significantly higher than those of the control group (*p* < 0.05), and the smoking rate was lower. This study confirmed that structured behavioral intervention can effectively promote high-risk populations to participate in screening and health management (65).

### 5.2 Strategies to improve screening adherence

Effective intervention measures include: health education supported by lectures or information technology; narrative intervention as a new and effective method; content covering cancer epidemiology, risk factors, early warning symptoms, and screening methods. All interventions have been proven to improve individuals’ knowledge level, screening willingness, and actual behavior, providing references for designing special interventions for gastric cancer (66).

## 6 Gastric cancer interventions and the correlation with clinical nutrition

### 6.1 AI-assisted diagnosis and treatment and its connection with clinical nutrition

AI plays an important role in gastric cancer diagnosis and treatment decision-making (67). In endoscopic detection, deep learning-based AI systems can realize real-time detection (CADe) and feature description (CADx) during gastrointestinal endoscopy (68), effectively identifying early gastric cancer and precancerous lesions, and guiding targeted biopsy, with diagnostic accuracy comparable to or even exceeding that of human endoscopists (69). In clinical decision support, systems such as GC-CDSS (70), MuMo model (71), iSCLM (72), and XGBoost complication prediction model can provide personalized treatment recommendations, predict treatment efficacy and postoperative complications, and improve the standardization and efficiency of diagnosis and treatment (73, 74). The application of AI is closely associated with clinical nutrition. For example, the XGBoost complication prediction model, which can assess the risk of postoperative complications in gastric cancer patients^75,^ has been further optimized by integrating nutritional indicators such as neutrophil-lymphocyte ratio (NLR), platelet-lymphocyte ratio (PLR), and skeletal muscle index (SMI). Nutritional intervention guided by AI prediction can effectively reduce the incidence of postoperative complications by improving the nutritional status of high-risk patients before surgery (70).

### 6.2 Surgical approaches and nutritional management for gastric cancer patients

In surgical approaches, Da Vinci robotic gastrectomy has advantages over laparoscopic gastrectomy in reducing intraoperative blood loss, shortening postoperative hospital stay, and reducing complication rates (75, 76). Clinical nutrition support is crucial for surgical patients: preoperative nutritional reserve can enhance the body’s tolerance to surgical trauma, while postoperative phased nutritional management (from clear liquid diet to soft diet) can promote gastrointestinal function recovery, accelerate wound healing and reduce the incidence of complications (77). For example, patients undergoing robotic Roux-en-Y Gastric Bypass (RYGB) can reduce the risk of anastomotic stenosis through standardized postoperative nutritional intervention, in addition to the advantages of surgical techniques (78).

### 6.3 Clinical nutrition of immunotherapy and targeted therapy

Precision targeted agents—including trastuzumab (HER2 inhibitor) (79, 80), ramucirumab (anti-angiogenic), and zolbetuximab (targeting Claudin 18.2) can precisely target tumor cells, improving treatment efficacy while reducing damage to normal tissues (81–83). However, these drugs may cause gastrointestinal reactions such as nausea and vomiting, leading to decreased appetite and nutritional intake. Clinical nutrition intervention can alleviate these adverse reactions through measures such as small frequent meals, easily digestible diet, and appropriate nutritional supplements, ensuring adequate energy and nutrient supply for patients, maintaining muscle mass (84).

In immunotherapy, PD-1/PD-L1 antibodies (such as nivolumab and pembrolizumab) and novel immune combination strategies (such as immunotherapy combined with chemotherapy) can activate the body’s immune system to eliminate tumor cells, showing significant efficacy in MSI-H/dMMR gastric cancer patients and advanced gastric cancer patients (85–87). Clinical nutrition can regulate the body’s immune function: adequate intake of high-quality protein, vitamins (such as vitamin C, D), and trace elements (such as zinc, selenium) can enhance immune cell activity, improve the immune response to immunotherapy (88). For example, the PD-1/CTLA-4 dual-target antibody combined with chemotherapy scheme, which has shown good efficacy in advanced gastric cancer, can further improve the therapeutic effect by matching nutritional support to enhance the body’s immune function (89).

## 7 Future challenges and research directions

### 7.1 Technical challenges

Despite the rapid progress of AI in nutrition and diagnosis, it still faces many challenges: insufficient model transparency, making it difficult to explain why specific recommendations are more effective for individuals (90); data inaccuracies caused by recall bias, intentional underreporting, and food classification and cultural differences (91); prominent data privacy and ethical issues arising from the use of genetic and microbiome data (92); lack of long-term follow-up data, making it difficult to confirm whether AI interventions can truly reduce cancer incidence (3). For example, AI systems for diabetic patients often struggle to meet specific nutritional needs, such as accurate carbohydrate intake or sodium intake restrictions (93).

### 7.2 Population differences and equity issues

Patients in comprehensive cancer centers have higher dietary quality and lower food insecurity than those in internet hospitals (94), indicating that the type of medical institution is an important factor affecting the nutritional status of cancer patients. Patients living in Food Insecure Areas (FPAs) have a 3-fold higher risk of food insecurity, higher unmet nutritional needs, and significant differences in cancer risk among residents in different residential areas (95). Within one year after gastric cancer surgery, female patients have significantly lower intake of energy, protein, and fat than male patients, suggesting that personalized nutritional support targeting different genders is needed during postoperative rehabilitation (96). A multi-ethnic cohort study found that higher overall dietary quality is associated with a lower risk of gastric cancer. Immigration studies have shown that individuals born in Japan have an increased risk of non-cardia gastric cancer, while no significant association was found in Korean or Chinese immigrants (2). IL-17 gene polymorphisms are significantly associated with gastric cancer risk in Han Chinese and Japanese populations, indicating the influence of genetic background (97). This requires future intervention strategies to consider diversity in ethnicity, culture, and dietary habits, avoiding “one-size-fits-all” recommendations.

### 7.3 Future research directions

An important direction for future research is to use AI to explore the complex impacts of multi-dimensional lifestyle factors such as diet and chronic diseases on the treatment and prognosis of gastrointestinal tumors (98). Specifically, algorithms such as Self-Organizing Map (SOM) clustering and Isolation Forest can be used to model and analyze dietary habit data to predict the risk of gastrointestinal tumor occurrence (99). Furthermore, by integrating multi-modal data such as endoscopic images, pathological sections, genomics, and diet, more powerful prediction models can be constructed, which will be an inevitable trend to achieve precise risk early warning and personalized nutritional intervention for gastric cancer and other gastrointestinal tumors (100). A critical priority is prospective trials testing AI dietary interventions in GC high-risk populations, with endpoints including GC incidence, precancerous lesion progression, and validated biomarkers. Without such evidence, current promise remains hypothetical. Meanwhile, with the deepening clinical application of AI, ensuring its safety and ethical compliance has become an urgent and critical issue to address. Future research should also focus on developing interpretable AI models (101), establishing standardized data privacy protection protocols, and algorithm audit mechanisms (102).

## 8 Conclusion

AI-assisted personalized dietary intervention represents a promising but unproven approach for GC prevention. Evidence from metabolic diseases demonstrates technological feasibility, but direct GC-specific trials are absent. Existing evidence shows that by integrating multi-dimensional information such as biomarkers, genetic information, dietary behavior, and microbiome data, AI models can play an important role in multiple links including gastric cancer risk prediction, personalized nutritional advice, healthy behavior guidance, early diagnosis, and even treatment decision-making, promoting the transformation of intervention strategies from “population guidelines” to “individual prescriptions.” From risk assessment based on the G-score model, intervention based on the Behavior Change Wheel (BCW) theory, to deep learning-assisted endoscopic detection and personalized clinical decision support systems (GC-CDSS), the entire prevention and control chain is gradually realizing intelligence and individualization. However, to achieve the widespread application of this technology, it is still necessary to overcome key challenges such as model transparency (interpretability), data security and ethics, verification of long-term intervention effects, and applicability across cultures and populations. Future research should prioritize rigorous clinical trials to validate these applications in high-risk populations and assess their long-term impact on gastric cancer outcomes.

## Methods

The literature discussed in this narrative review was identified through searches of PubMed and Web of Science using combinations of keywords related to Artificial Intelligence, Gastric Cancer, Personalized Diet and Risk Stratification. The primary focus was on peer-reviewed articles published within the past 1–6 years, with earlier seminal studies included when necessary to provide historical context and mechanistic foundations. No predefined inclusion or exclusion criteria or quantitative synthesis was applied, consistent with the narrative and integrative nature of this review.

## Glossary

AI: Artificial Intelligence
GC: Gastric Cancer
Hp: *Helicobacter pylori*
CAG: Chronic Atrophic Gastritis
IM: Intestinal Metaplasia
LGD: Low-Grade Dysplasia
HGD: High-Grade Dysplasia
GRAPE: Gastric Cancer Risk Assessment Program
HDGC: Hereditary Diffuse Gastric Cancer
GAPPS: Proximal Polyposis of the Stomach
FIGC: Familial Intestinal-Type Gastric Cancer
ML: Machine Learning
DL: Deep Learning
LLMs: Large Language Models
NLP: Natural Language Processing
IoT: hybrid systems combined with the Internet of Things
DII: Dietary Insulin Index
DIL: Dietary Insulin Load
CADe: Computer-Aided Detection
CADx: Computer-Aided Diagnosis
ADC: Antibody-Drug Conjugate
GC-CDSS: Gastric Cancer Treatment Recommendation System
PUFAs: Polyunsaturated Fatty Acids
BCW: Behavior Change Wheel
NLR: Neutrophil-Lymphocyte Ratio
PLR: Platelet-Lymphocyte Ratio
SMI: Skeletal Muscle Index
FPAs: Food Insecure Areas
